# Preparing to Meet the Needs of a Growing Older Adult Population with Type 1 Diabetes: A Narrative Review

**DOI:** 10.1007/s11606-025-10053-3

**Published:** 2025-12-08

**Authors:** Anna R. Kahkoska, Joshua J. Neumiller, Anastasia-Stefania Alexopoulos, Antoine Christiaens, Tali Cukierman-Yaffe, Nicole M. Ehrhardt, Elbert S. Huang, Thaer Idrees, Lori M. Laffel, Sei J. Lee, Naushira Pandya, Richard E. Pratley, Leocadio Rodriguez-Mañas, Christine Slyne, Elena Toschi, Ruth S. Weinstock, Adriana Wisniewski, Carol Wysham, Medha Munshi

**Affiliations:** 1 Division of Endocrinology and Metabolism, University of North Carolina at Chapel Hill, Chapel Hill, NC USA; 2 Department of Nutrition, Gillings School of Global Public Health, University of North Carolina at Chapel Hill, Chapel Hill, NC USA; 3 UNC Center for Aging and Health, University of North Carolina at Chapel Hill, Chapel Hill, NC USA; 4 Department of Pharmacotherapy, College of Pharmacy and Pharmaceutical Sciences, Washington State University, Spokane, WA USA; 5 Department of Medicine, Duke University School of Medicine, Durham, NC USA; 6 Clinical Pharmacy and Pharmoepidemiology Research Group, Louvain Drug Research Institute, Université Catholique de Louvain, Brussels, Belgium; 7 Fund for Scientific Research – FNRS, Brussels, Belgium; 8 Division of Endocrinology, Metabolism and Diabetes, Sheba Medical Center, Ramat Gan, Israel; 9 Department of Epidemiology and Preventative Medicine, School of Public Health, Faculty of Medicine, Herczeg Institute On Aging, Tel-Aviv University, Tel Aviv, Israel; 10 Department of Metabolism, Endocrinology and Nutrition, Medicine Diabetes Institute, University of Washington, Seattle, WA USA; 11 Department of Medicine, University of Chicago, Chicago, IL USA; 12 Emory University School of Medicine, Atlanta, GA USA; 13 Joslin Diabetes Center, Boston, MA USA; 14 Beth Israel Deaconess Medical Center, Boston, MA USA; 15 Harvard Medical School, Boston, MA USA; 16 Division of Geriatrics, School of Medicine, University of California, San Francisco, CA USA; 17 Department of Geriatrics, Dr. Kiran C. Patel College of Osteopathic Medicine, Nova Southeastern University, Fort Lauderdale, FL USA; 18 Advent Health Translational Research Institute, Orlando, FL USA; 19 Servicio de Geriatria, Hospital Universitario de Getafe, Getafe, Spain; 20 Department of Medicine, Upstate Medical University, Syracuse, NY USA; 21 Department of Anthropology, University of North Carolina at Chapel Hill, Chapel Hill, NC USA; 22 Section of Endocrinology and Metabolism, MultiCare Rockwood Clinic, Spokane, WA USA

**Keywords:** type 1 diabetes, older adults, internal medicine, geriatrics, endocrinology

## Abstract

The prevalence of diabetes is rising among older adults. While most diabetes cases in older adults are type 2 diabetes mellitus (T2D), advances in type 1 diabetes mellitus (T1D) management and rising rates of adult-onset T1D have translated into a growing number of individuals with T1D living into older adulthood. This narrative review integrates existing evidence on management of T1D in older adults with expert opinions to provide practical guidance for generalists increasingly encountering the unique challenges and complexities of this growing population. The profound heterogeneity in clinical presentation, pathobiology, and disease progression across the older adult population can make it challenging to differentiate older adults with T1D from those with insulin treated T2D, particularly in adult-onset cases. However, timely diagnosis is critical to minimize exposure to hyperglycemia and reduce the risk for complications, as individuals with T1D rely entirely on exogenous insulin and require intensive self-management to prevent acute complications like hypoglycemia and ketoacidosis. Self-management of T1D in older adults presents unique challenges, including a high risk of hypoglycemia that must be mitigated in the setting of a lifelong requirement for insulin and evolving mismatches between intensive self-management demands and an older person’s capacity for self-care. The growing number of older adults with T1D underscores a lack of access to specialized care and limited training and resources for evidence-based management in primary care and post-acute/long-term care settings, as well as the dearth of high-quality clinical evidence specific to this population to inform care. Research to support changes across healthcare systems and at the policy level, in combination with education and multi-specialty collaboration, will ensure that healthcare providers and health systems are equipped and prepared to better meet the needs of the growing population of older adults with T1D.

**Supplementary Information:**

The online version contains supplementary material available at 10.1007/s11606-025-10053-3.

## INTRODUCTION

In the United States (US), approximately 25% of individuals ≥ 65 years old have diabetes.^1^ As this number is projected to increase, so too does the strain on healthcare systems; in 2022, approximately 67% of US healthcare expenditures for diabetes were incurred by individuals aged ≥ 65 years.^2^ Similar trends have been observed in European countries.^3^

Type 2 diabetes (T2D) comprises most diabetes cases in older adults. However, with advances in care, an increasing number of individuals with type 1 diabetes (T1D) are now living into older adulthood, and it is increasingly recognized that T1D can be diagnosed at any age.^4–7^ The increasing prevalence in older adults is a testament to the successes of modern medicine,^8,9^ yet this demographic shift presents new challenges for healthcare providers and systems. For example, whereas some older adults with T2D may require insulin and glucose monitoring, those with T1D require continuous exogenous insulin replacement and intensive self-management to prevent acute complications, including severe hypoglycemia and diabetic ketoacidosis (DKA).

This narrative review aims to summarize existing evidence on older adults with T1D and provide practical guidance for generalists increasingly encountering the unique challenges and complexities of this growing population.

## METHODS OF THE REVIEW

The multidisciplinary author team includes endocrinologists, geriatricians, internists, pharmacists, and clinical, epidemiology, and health services researchers. The authors gathered in Boston at an International Geriatric Diabetes Society (IGDS) Workshop on Type 1 Diabetes in Older Adulthood from September 4–6, 2024. Workshop participants (clinicians, researchers, industry partners, persons with diabetes, and their care partners) engaged in discussions related to the diagnosis and management of T1D in older adults, including challenges faced from patient/provider perspectives, recommendations for care, and identification of critical knowledge gaps.

The workshop and subsequent discussion helped to guide the organization of the manuscript, the selection of sections, identification of key data sources, and recognition of important gaps. To augment research and discussions from the workshop, we evaluated the evidence base for peer-reviewed manuscripts on focused topics relevant to older adults with T1D: (1) qualitative studies; (2) frailty; (3) cognitive impairment. We developed a search strategy for each topic to identify eligible studies using a combination of Medical Subject Headings (MeSH) and text words in PubMed (U.S. National Library of Medicine, National Institutes of Health); the search terms are summarized in Supplemental Table S1. We included only peer-reviewed publications, studies published in English, and those with publication dates from 11/2004–11/2024. We included studies of older adults (mean age ≥ 65; none < 60 years) with T1D. We excluded studies with young or middle-aged adults or individuals with type 2 diabetes. We omitted searching grey literature, conference abstracts, and trial registry websites. All searches were executed on November 15, 2024, in PubMed. A total of 182 full-text articles were reviewed across three focus areas—qualitative studies, cognitive impairment, and frailty—of which 28 were included in the narrative review after excluding 154 for lack of relevance (Supplemental Table S2). Clinical guidelines and expert consensus reports were reviewed separately by a team of clinical experts for their key statements relevant to older adults with T1D. Given the lack of data in older adults with T1D, national policy documents and clinical guidelines have largely offered recommendations focused on management and care of older adults with T2D. The author team has synthesized policy and guidelines pertaining to older adults with T1D where and when available.

## EPIDEMIOLOGY OF TYPE 1 DIABETES IN OLDER ADULTHOOD

In adults aged ≥ 65 years, T1D prevalence increased by 180% between 1990 and 2019, with 3.7 million now affected worldwide.^7^ T1D prevalence varies based on world region and country-level economic status, and there are high rates of misclassification with type 2 diabetes.^6^ However, it is estimated that ~ 16% of prevalent T1D cases globally are in the ≥ 65 age group,^10^ and in high-income countries, older adults are estimated to make up 18% of the population with T1D.^11^ In the US, the prevalence of T1D in older adults (≥ 65 years) between 2019–2022 was 5.3 per 1000 adults (95% CI 4.4–6.2), but highest among middle-aged adults (45–64 years) at 6.1 (95% CI 5.2–7.1) compared to both the younger and older adults.^12^

The rising prevalence partly reflects increasing life expectancy in people with T1D,^13,14^ resulting in a greater number of people living into older adulthood years. Although life expectancy in T1D was reported lower compared with the general population without diabetes in a Scottish cohort study,^15^ it may vary drastically according to birth cohort^14^ and other modifiable risk factors, such as smoking and body mass index.^16^ A second contributing factor is the rising incidence of T1D diagnoses in adulthood.^7,17^ Global data from 2021 indicated that the number of incident T1D cases was numerically higher in adults than in children (316,000 vs. 194,000).^17^ In a large US-based cohort study of over 1,000 adults with T1D, 23.8% were diagnosed after the age of 30 (range 18–78).^18^ The Type 1 Diabetes Exchange Registry and online patient community also conducted an exploratory study about the age at time of diabetes diagnosis, with over 3,000 participant responses (800 diagnosed in adulthood),^19^ where the average age at diagnosis was 31 years, with a range of 18 to 74 years.^6^ Although survival has improved for older adults with T1D, they still have a lower life expectancy compared with their peers without diabetes.^6^

## DIAGNOSIS OF TYPE 1 DIABETES IN OLDER ADULTS

Complexities surrounding discerning diabetes type among older adults apply to epidemiologic research and clinical settings. T1D and T2D are heterogeneous diseases with significant variability in clinical presentation and disease progression, particularly due to residual endogenous insulin production that is often present in people diagnosed with T1D in adulthood.^20^ Adult-onset disease may exhibit diverse metabolic abnormalities at diagnosis, ranging from diabetic ketoacidosis (DKA) to mild non-insulin-requiring diabetes.^4,21^ Further, the rate of progression to severe insulin deficiency and initial diagnosis in adult-onset T1D varies significantly, particularly at an older age, further obscuring accurate classification between type 1, type 2, and other types of diabetes.^5,22^

One barrier to accurate diagnosis among adult-onset cases may be the historical perspective that T1D is a childhood disease. Self-reported data from a US-based online survey study suggested that a true T1D diagnosis was missed in 16% of those diagnosed before the age of 18 and in 38.6% of those diagnosed at 18 years or older.^19^ Among individuals over 30 years old, 46% were misdiagnosed, rising to 48% among those over 40, and reaching 55% for those over 50 years old at time of diagnosis.^19^

Additionally, older adults with T1D can have obesity and insulin resistance along with residual beta cell function,^20^ thus sharing attributes of the T2D phenotype. They may share metabolic comorbidities, including sleep apnea, metabolic-associated liver disease, and cardiovascular disease.

The complexity surrounding accurate diagnosis of diabetes in older adults can be further complicated by other etiologies and risk factors. Immune checkpoint inhibitors (ICI), including anti–cytotoxic T lymphocyte antigen 4 (CTLA-4), anti–programmed cell death 1 (PD-1), and anti–programmed cell death ligand 1 (PD-L1) antibodies, are currently administered to older adults to treat malignancies, including lung cancer and melanoma. Unfortunately, treatment with these agents can lead to autoimmune endocrinopathies, with ICI-induced T1D (ICI-T1D) now observed in up to 1.4% of patients receiving these potentially life-saving medications according to an analysis of a large U.S. commercial health insurance claims database.^23^ Moreover, recent data have highlighted the association with new-onset T1D following a Coronavirus Disease 2019 (COVID-19) infection, revealing heightened risk among older adults specifically.^24^ Finally, type 3c diabetes, also known as pancreatogenic diabetes, occurs due to conditions such as acute and chronic pancreatitis, pancreatic cancer, and pancreatic surgery and accounts for 1–9% of all diabetes cases.^25^ Older adults are particularly susceptible due to a higher prevalence of underlying pancreatic conditions compared with younger adults.^25^

The primary risk associated with severe insulinopenia and misdiagnosis is prolonged exposure to hyperglycemia, which can increase the risk for chronic complications, as well as DKA, which is associated with high morbidity and mortality.^4^ Misdiagnosis may be mitigated with ongoing evaluation of diabetes type, for example, with autoantibody testing and awareness of progression to insulinopenia, particularly for older adults experiencing worsening glycemia.

## IMPACTS OF AGE-RELATED CONDITIONS ON TYPE 1 DIABETES SELF-MANAGEMENT

The older adult population with diabetes is heterogeneous in physical, functional, and cognitive health.^9,26^ While some older adults with diabetes remain independent and healthy, others may experience frailty, cognitive decline, and multiple comorbidities. Furthermore, social and environmental factors, such as living arrangements, access to healthcare, and family support, play a critical role in shaping care needs.^9^

An important consideration is that all older adults with T1D require lifelong insulin therapy that must be sustained in the face of comorbidities that complicate insulin administration and safety, such as dexterity issues, cognitive decline, and visual impairments. To maintain blood glucose within target ranges, individuals living with T1D are required to inject or bolus the appropriate amount of insulin at or just before meals, taking into consideration carbohydrates ingested and ambient glucose levels. They are also required to continually adjust doses to accommodate activity levels, stress, and illness, manage technology, and react to hypoglycemia and hyperglycemia.

Advanced diabetes technologies, including insulin pumps, continuous glucose monitors (CGM), and automated insulin delivery (AID) systems, are now recommended as standard of care for T1D management across the lifespan, as long as there are no safety concerns.^27^ Randomized clinical trials enrolling older adults have demonstrated that both CGM and AID reduced the risk of hypoglycemia in this age group.^28–34^ However, there are known age-related barriers to adoption and sustained use, which is dependent upon comfort adapting to new technology,^35^ cognitive and physical abilities,^36^ access to appropriate training programs,^35^ availability of adequate training and care partner support, ^37,38^ financial concerns,^39,40^ and the usability of device components for older adult populations, especially for those in assisted living or nursing facilities.^35,39,41,42^

**Figure 1:**
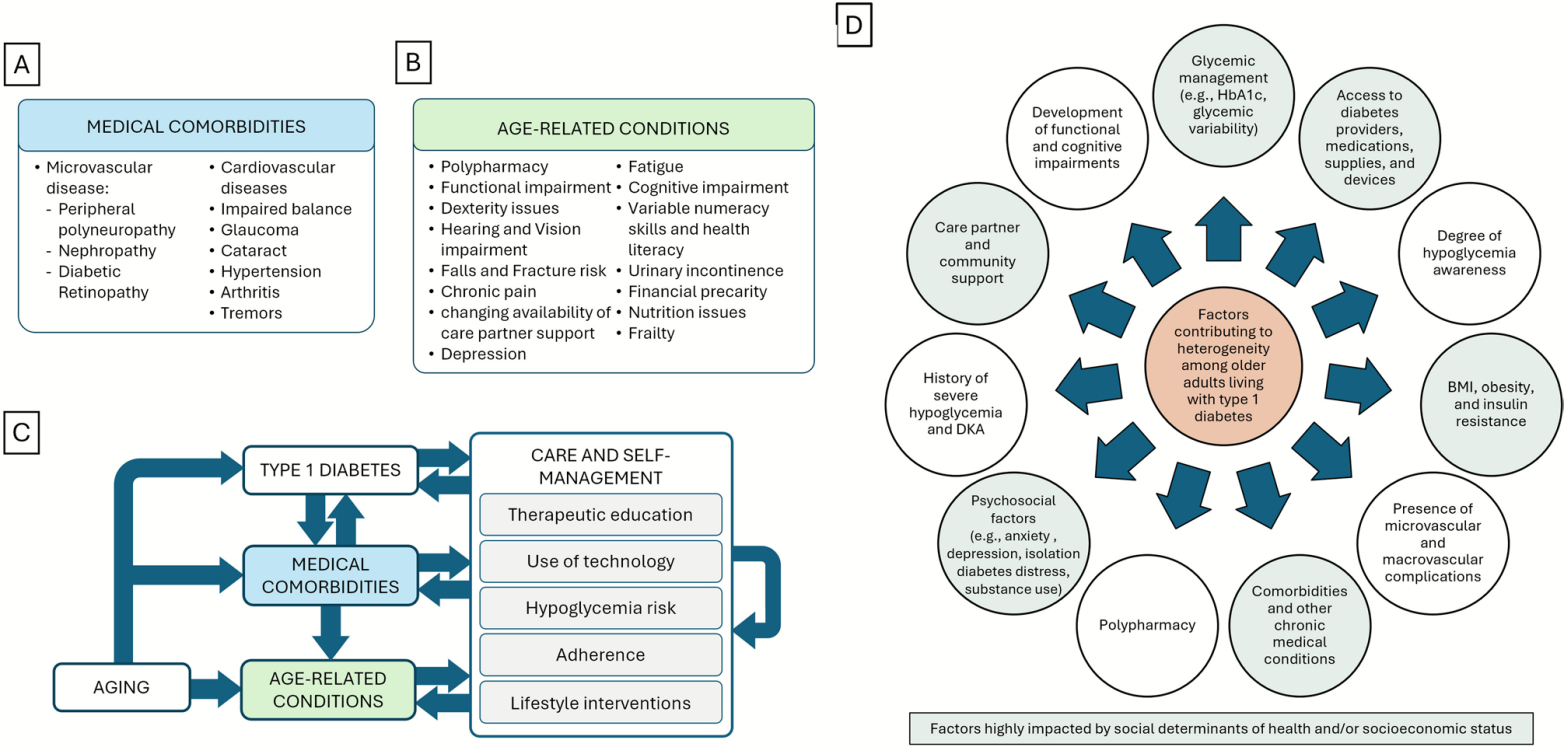
Interactions between medical comorbidities, age-related conditions, and care and self-management challenges in older adults living with type 1 diabetes (T1D). Cognitive impairment, hearing and vision loss, or lacking care partner support may impair nearly all dimensions of self-management. Conversely, certain management domains are heavily impacted by age-related conditions, such as the ability to use diabetes technology, which is affected by factors like dexterity issues, functional impairment, financial insecurity, cognitive decline, and hearing and vision impairments. (**A**) List of common medical comorbidities associated with aging and T1D in older adults; (**B**) List of age-related conditions in older adults living with T1D; (**C**) Pathways showing how T1D, medical comorbidities, and age-related conditions interact and potentially affect care and self-management domains; (**D**) Factors contributing to heterogeneity among older adults living with T1D.

Together, managing T1D in older adults is challenged by potential interactions between medical comorbidities (Fig. 1A) and age-related conditions (Fig. 1B). Comorbidities, resulting from the accumulation of conditions over time—directly caused by T1D or not (e.g., microvascular complications – including diabetic retinopathy, peripheral neuropathy, diabetic nephropathy, cardiovascular diseases, depression)^43,44^ – exacerbate the development of age-related conditions (e.g., cognitive impairment, dexterity issues, vision and hearing impairment) arising from pathological aging processes (Fig. 1C).^45^ In turn, age-related conditions significantly disrupt multiple aspects of T1D self-management, including engagement with lifestyle interventions (e.g., physical activity, nutrition), diabetes technology, hypoglycemia risk management (including hypoglycemia awareness), treatment adherence, and understanding and receiving diabetes education (Fig. 1C).^46^ Suboptimal care and self-management can increase risk of hypoglycemia, and hyperglycemia which may accelerate cognitive decline and worsen comorbidities.^45,47–49^ These challenges may be compounded over time by changes in care partner support, particularly when there is declining or limited availability of tactical, emotional, or informational assistance from spouses, adult children, or other care partners.

In particular, while the risk for cognitive impairment increases with age for all adults, people with T1D have an increased risk for dementia, structural brain abnormalities, an accelerated rate of cognitive decline;^34^ as well as, increased risk of cognitive decline with higher HbA1c levels, more episodes of severe hypoglycemia, and elevated systolic blood pressure.^35^ Recent studies of older adults with T1D highlighted that 48% exhibited clinically significant cognitive impairment,^36^ which is a higher rate than those without diabetes, and those with T1D had lower cognitive scores in language, verbal episodic memory, and executive function/psychomotor processing speed than compared with those with type 2 diabetes or those without diabetes.^37,50–53^ History of recurrent severe hypoglycemia,^35,36,38–41^ chronic hyperglycemia,^39^ and diabetic ketoacidosis,^42^ are associated with an acceleration in cognitive decline.^51,54–57^ Younger age at diagnosis, longer disease duration, sustained hyperglycemia,^42^ high systolic blood pressure,^35^ increased lipids, impaired kidney function, and other diabetes-related complications also increase risk for cognitive impairment in T1D.^51,58,59^

Cognitive impairment and diabetes outcomes have a bidirectional nature (Fig. 1C); older adults with T1D and cognitive impairment may face difficulties with self-management, especially related to glucose monitoring and insulin management, inconsistent meal consumption, and making lifestyle adjustments.^60^ However, cognitive decline directly complicates insulin administration, leading to potential under- or over-dosing and increasing the risk for hyper- and hypoglycemia. Cognitive impairment may also impact digital health literacy and ability to utilize diabetes technologies safely and effectively.

## CLINICAL RECOMMENDATIONS FOR OLDER ADULTS LIVING WITH TYPE 1 DIABETES

Many factors contribute to the heterogeneity of older adults with T1D, and decision-making when considering therapeutic options, as shown in Fig. 1D. As the number of people with T1D living into older age increases, professional organizations are beginning to offer guidance and considerations for optimized management of this growing population.^61^ However, there is a lack of specific robust recommendations for the treatment of T1D in older adults, which largely reflects the lack of high-quality clinical evidence and randomized trials enrolling this population.

### Outpatient Care

Management of T1D in the outpatient setting requires careful consideration of person-specific (e.g., personal preferences, self-care capabilities, comorbidities, and complications) and environment-specific factors (e.g., financial and insurance considerations, living arrangements, care partner support) to ensure the treatment approach can be reasonably and safely implemented and maintained. However, guideline recommended basal-bolus insulin treatment regimens have a narrow therapeutic window that can result in both hypoglycemia and hyperglycemia, and the consequences of severe events (e.g., falls, fractures, arrhythmias, and cognitive impairment) may produce more harmful impacts in older age.

Table 1 summarizes key recommendations informing the care of older adults with T1D in the outpatient/ambulatory care setting. Given that the large majority of older adults with diabetes have T2D, combined with the lack of evidence from well conducted studies in older adults with T1D, most clinical practice guidelines and policy statements published to date focus on the T2D population. For these reasons, there exist large gaps in guidance for management of older adults with T1D, as discussed later. Where guidance is available, however, the importance of balancing glycemic goals and treatment approaches with the presence of key comorbidities and geriatric syndromes is stressed.^9,62^ They also underscore the value of person-centered education, and optimizing use of technology to improve insulin use safety and prevent severe hypoglycemia.^9,62^ Some guidelines provide recommendations with respect to treatment of all types of diabetes in older age,^9,63,64^ while others provide guidelines with respect to treatment of T1D in all age groups.^65,66^

**Table 1:** Summary of Key Recommendations Informing Care of Older Adults with Type 1 Diabetes in the Ambulatory Care Setting

Guideline/Position Statement	Select Recommendations/Statements
2025 American Diabetes Association Standards of Care in Diabetes^5^	*Balancing Diabetes Management with Comorbidities* • Consider the assessment of medical, psychological, functional (self-management abilities), and social domains in older adults with diabetes to provide a framework to determine goals and therapeutic approaches for diabetes management • Screen for geriatric syndromes (e.g., cognitive impairment, depression, urinary incontinence, falls, persistent pain, and frailty) and polypharmacy in older adults with diabetes, as they may affect diabetes self-management and diminish quality of life *Cognition Assessment* • Screening for early detection of mild cognitive impairment or dementia should be considered for adults 65 years of age and older at the initial visit, annually, and as appropriate *Treatment* • Simplification of complex treatment plans (especially insulin) is recommended to reduce the risk of hypoglycemia and polypharmacy and decrease the treatment burden if it can be achieved using the individualized glycemic goals *Technology* • For older adults with T1D, CGM is recommended to reduce hypoglycemia • For older adults with T1D, consider use of AID systems and other advanced insulin delivery devices such as connected pens to reduce risk of hypoglycemia, based on individual ability and support system *Nutrition & Physical Activity* • Optimal nutrition and protein intake is recommended for older adults with diabetes; regular exercise, including aerobic activity, weight-bearing exercise, and/or resistance training, should be encouraged in all older adults with diabetes who can safely engage in such activities
Endocrine Society Clinical Practice Guideline on Treatment of Diabetes in Older Adults^54^	*Balancing Diabetes Management with Comorbidities* • In adults aged 65 years and older with diabetes, assess overall health and personal values prior to the determination of treatment goals and strategies *Cognition Assessment* • In adults aged 65 years and older with diabetes, perform periodic cognitive screening to identify undiagnosed cognitive impairment • In adults aged 65 years and older with diabetes and a diagnosis of cognitive impairment, we suggest that medication regimens should be simplified and glycemic targets tailored to improve adherence and prevent treatment-related complications *Technology* • In adults aged 65 years and older with diabetes who are treated with insulin, we recommend frequent fingerstick glucose monitoring and/or CGM in addition to A1C *Nutrition* • In adults aged 65 years and older with diabetes, we recommend assessing nutritional status to detect and manage malnutrition *Frailty* • In adults aged 65 years and older with diabetes and frailty, we suggest the use of diets rich in protein and energy to prevent malnutrition and weight loss
NICE Quality Standard for Type 1 Diabetes in Adults^55^	*Education* • *Quality Statement*: Adults with T1D are offered a structured education program *Technology* • *Quality Statement:* Adults with T1D are offered use of CGM
Endocrine Society Clinical Practice Guideline on Management of Individuals with Diabetes at High Risk for Hypoglycemia^56^	*Education* • We recommend that a structured program of patient education over unstructured advice be used for adult outpatients with T1D *Treatment* • We recommend CGM rather than blood glucose monitoring (BGM) by fingerstick for adults with T1D receiving multiple daily injections • We suggest long-acting insulin analogs be used rather than NPH insulin for adult outpatients on basal insulin therapy who are at high risk for hypoglycemia • We suggest that rapid-acting insulin analogs be used rather than regular human insulin for adult patients on basal-bolus insulin therapy who are at high risk for hypoglycemia *Hypoglycemia Treatment* • We recommend that glucagon preparations that do not have to be reconstituted over glucagon preparations that do have to be reconstituted (i.e., available as a powder and diluent) be used for outpatients with severe hypoglycemia
NICE Quality Standard for Type 1 Diabetes in Adults: Diagnosis and Management^57^	*Balancing Diabetes Management with Comorbidities* • Take account of any disabilities, including visual impairment, when planning and delivering care for adults with T1D • View each adult with T1D as an individual, rather than as a member of any cultural, economic or health-affected group *Education* • Advice to adults with T1D should be provided by a range of professionals with skills in diabetes care, working together in a coordinated approach • Offer all adults with T1D a structured education program of proven benefit *Technology* • Offer adults with T1D a choice of real-time or intermittently scanned CGM, based on their individual preferences, needs, characteristics, and the functionality of the devices available *Nutrition & Physical Activity* • Provide nutritional information individually and as part of a structured education program • Modify nutritional recommendations to adults with T1D to take account of associated features of diabetes, including: excess weight and obesity, underweight, disordered eating, hypertension, and renal failure For adults with T1D who choose to increase their level of physical activity as part of a healthier lifestyle, provide information about appropriate intensity and frequency, self-monitoring their changed insulin and/or nutritional needs, effect of physical activity on blood glucose levels, and appropriate adjustments of insulin dosage or nutritional intake
AACE Guideline on Use of Advanced Technology in the Management of Persons with Diabetes^58^	*Technology* • CGM is strongly recommended for all persons with diabetes treated with intensive insulin therapy, defined as 3 or more injections of insulin per day or the use of an insulin pump • Connected pens may be recommended for all persons with diabetes who are treated with intensive insulin management, with 3 or more injections per day and who are not on insulin pump therapy, in whom an assessment of insulin dosing may help the person with diabetes and the clinician to further optimize the insulin regimen and avoid the stacking of rapid-acting insulin doses that could lead to hypoglycemia • AID systems are strongly recommended for all persons with T1D • Clinicians should strongly consider the discontinuation of insulin pump therapy based on an individual’s ability to use it effectively and safely or based on the personal preference of a person with diabetes to discontinue this insulin delivery modality

Abbreviations: AACE, American Association of Clinical Endocrinology; A1C, glycated hemoglobin A1c; ADA, American Diabetes Association; AID, automated insulin delivery; CGM, continuous glucose monitoring; NICE, National Institute for Health and Care Excellence; NPH, Neutral Protamine Hagedorn; BGM, blood glucose monitoring; T1D, type 1 diabetes

### Transitions of Care and Post-Acute Settings

Table 2 summarizes key recommendations informing the care of older adults with T1D in settings outside the home. Older adults with T1D require a multidisciplinary care approach for glucose management and education during hospitalization. It is essential to provide basal insulin coverage even when patients are required to abstain from eating and drinking prior to procedures (i.e., Nothing by mouth (NPO)), and staff education should include training on insulin pumps, CGM, carb counting and recognition and treatment of hypoglycemia. During hospitalizations, transitioning from intravenous to subcutaneous insulin requires knowledgeable and experienced care providers to ensure sufficient overlap of insulin action to avoid complications such as DKA or rebound hyperglycemia. In non-critically ill patients, the continuation of CGM and AID systems can help manage glycemic variability, though these devices are not U.S. Food and Drug Administration (FDA)-approved for hospital use and require knowledgeable hospital staff for their successful inpatient implementation. In long-term care settings, glucose management should be individualized, avoiding reliance on HbA1c and focusing on minimizing both hypoglycemia and symptomatic hyperglycemia.^18,49,67–74^ In end-of-life care, the focus should be on comfort, reducing insulin regimens to basal insulin when appropriate, and avoiding strict glycemic control.^73,74^

**Table 2:** Summary of Key Recommendations Informing Care of Older Adults with T1D in Environments Specific to Older Adults Outside of the Home

Location	Select Recommendations/Statements
Hospitalization^60,62,63,65,68–71^	*General approaches:* • Implement multidisciplinary care for glucose management and education upon admission for better outcomes • Screen for frailty and consider admission to ACE units^72,73^ *Staff Education:* • Older adults with T1D need basal insulin coverage even when they have no oral intake • Consider teaching nursing staff about carb counting • CGM and insulin pumps basics (if applicable) *Management:* • Transition to subcutaneous insulin from intravenous requires 2–4 h of overlap to prevent recurrence of DKA or rebound hyperglycemia • Encourage the continuation of CGM and AID upon hospital admission to reducing glycemic variability and hypoglycemia in selected, noncritically ill patients (CGM and AID are not FDA approved to manage DM in hospital) + BGM confirmation using hospital-calibrated glucometer • Assure enough supplies for AIDs or CGM • Check infusion/injection sites in hospital • Avoid SGLT2i if admitted with HF • Falls precautions protocol • Initiation of physical therapy/walks *Screen daily for:* • Erratic food intake, TF/TPN changes, • Medications affecting BG (e.g., corticosteroids, antipsychotics, antiretroviral therapy • Renal function • Dextrose containing fluids *Diet:* • High protein carb-controlled diet and eliminate sweetened beverages *Insulin orders:* • Avoid reliance on sliding scale regular insulin • Consider insulin drip for acutely ill patients in the ICU and post-surgery and avoid subcutaneous insulin, in particular during hypotension/shock • Consider starting total daily dose (TDD) at 0.2 to 0.3 units/kg per day • Insulin adjustments if BG < 100 mg/dL • Prandial insulin after meals for unpredictable oral intake *Glucose targets:* 140–180 mg/dL while avoiding hypoglycemia in the hospital, but can accept up to 250 mg/dL in: • Advanced CKD • High risk for hypoglycemia • Brittle diabetes with significant glycemic excursions • No BG targets are established for rehabilitation, consider individualization of therapy according to frailty/functional level *Vision aids:* • Discharge on insulin pen with teaching ± magnifier or “talking” glucose meter • teach counting clicks for dosing
Skilled rehabilitation (SNF)^18^	*General approach:* • Comprehensive assessment of comorbidities, mentation, mobility, medications (insulin regimen and BG monitoring), and goals of care • Evaluate potential for discharge, caregiver and community support • Evaluate self-care and functional ability • Providers and institutions to standardize system and parameters for glucose emergencies communications • BG monitoring frequency; 3 to 5 times a day (AC and HS or PPG and in the middle of the night if indicated) if no CGM • Consult endocrinologist if BG control is suboptimal or patient has complex insulin delivery system *Staff and Practitioner Education:* • Older adults with T1D need basal insulin coverage even when NPO • CGM and insulin pumps basics (if applicable, and was used prior to SNF transfer) • Insulin requirements may increase during infections, cardiovascular events and other emergencies • Recognition and treatment of hypoglycemia *Treatment targets:* • Avoid reliance on A1C due to acute illness • Fasting and pre-meal BG targets 100–200 mg/dL (5.6–11.1 mmol/L) • Discontinue sliding scale insulin administration *Screen for:* • Renal insufficiency • Irregular meal intake and weight loss • Depression and cognitive impairment (affect self-care) • Foot problems *Insulin order:* • Administer prandial insulin immediately after meals for unpredictable oral intake • Avoid NPH/Regular insulins due to higher risk of hypoglycemia, or provide more frequent snacks • Recommended degludec U100 insulin or glargine U300 insulin for basal coverage • Avoid sliding scale use with regular insulin
Assisted living facilities^12,16,18^	*General approaches:* • Comprehensive assessment of comorbidities, mentation, mobility, medications (insulin regimen and BG monitoring), and goals of care • Evaluate diabetes self-management ability • Assess staffing capability (BG monitoring and injections) • Assess engaged caregiver and community support NOTE: Care of T1D patients in this setting is usually not feasible without competent patient or caregiver
Long term care facilities (LTCF) ^18,44,59–66^	*General approaches:* • Comprehensive assessment of comorbidities, mentation, mobility, medications (insulin regimen and BG monitoring), and what matters most (goals of care) utilizing the 4 M approach to age friendly health care • Providers and institution to standardize system and parameters for glucose emergencies communications • Treatment can be adjusted remotely *Staff and Practitioner Education:* • Older adults with T1D need basal insulin coverage even when NPO • CGM and insulin pumps basics (if applicable, and was continued in LTCF) • Insulin requirements may increase during infections, cardiovascular events and other emergencies • Recognition and treatment of hypoglycemia (especially if cognitively impaired) *Targets:* 100–200 mg/dL on BGM, or TIR > 50%, with TBR < 1% on CGM ^74^ • Providers to individualize targets according to functional status and life expectancy and not based on age • Less reliance on A1c • Avoid hypoglycemia and symptomatic hyperglycemia • Discontinue sliding scale insulin administration *Screen for:* • Cognitive impairment and medications that mask hypoglycemia awareness • Chronic pain • Polypharmacy and potential to simplify or deintensify treatment regimen • Recurrent falls • Depression • Urinary incontinence (new or worsening) *Diet:* • Consider healthful diet with high protein *Insulin order:* • Administer prandial insulin immediately after meals for unpredictable oral intake • Avoid NPH/Regular insulins due to higher risk of hypoglycemia, or provide more frequent snacks • Recommended degludec U100 insulin or glargine U300 insulin for basal coverage • Avoid sliding scale with regular insulin *Other management:* • Encourage weightbearing and resistant exercises • Address hypertension and hyperlipidemia in individuals with higher risk for atherosclerotic cardiovascular disease and have longer life expectancy • BP targets: o Complex/Intermediate: < 140/80 mmHg, o Very complex/poor health: < 150/90 mmHg
Discharge Planning^18^	• Avoid medication intensification at hospital discharge • Involve caregiver in all discharge instructions • Screen for and address visual impairment, functional status to assess self-management abilities, that may impact self-management • Discuss goals of care • Review all medications (indications, doses, and changes) with patient and caregiver • Home health referral • Avoid medication intensification at hospital discharge • Provide after visit summary with big size font instruction • Provide talking glucometer if vision impairment is present • Assist in scheduling follow-up visits with primary clinician and selected specialists • Transfer hospital records or discharge summary to primary clinician • Assist patient and/or caregiver with access to medical records through patient portal if available
End of life^65,66^	*General approaches:* • Address goals and intensity of care with care partners • Avoid strict glucose and blood pressure management • No role for A1C measurements • Encourage simplification/reduction of insulin regimen possibly to basal insulin only • Goals are to prevent severe hypoglycemia and hyperglycemia to promote comfort and preserve quality of life

Abbreviations: A1C, glycated hemoglobin A1c; AC, before meals; AID, automated insulin delivery; BG, blood glucose; BGM, blood glucose monitoring; BP, blood pressure; CGM, continuous glucose monitoring; CKD, chronic kidney disease; DM, diabetes mellitus; FDA, U.S. Food and Drug Administration; HF, heart failure; HS, at bedtime; LTCF, long-term care facility; NPH, Neutral Protamine Hagedorn; NPO, nothing by mouth; PPG, postprandial glucose; SGLT2, sodium-glucose co-transporter 2; SNF, skilled nursing facility; T1D, type 1 diabetes; TBR, time below range (< 70 mg/dL); TF/TPN, tube feeding/total parenteral nutrition; TIR, time in range (70–180 mg/dL); 4 M, mentation, mobility, medications, and what matters most

## DISCUSSION

**Figure 2:**
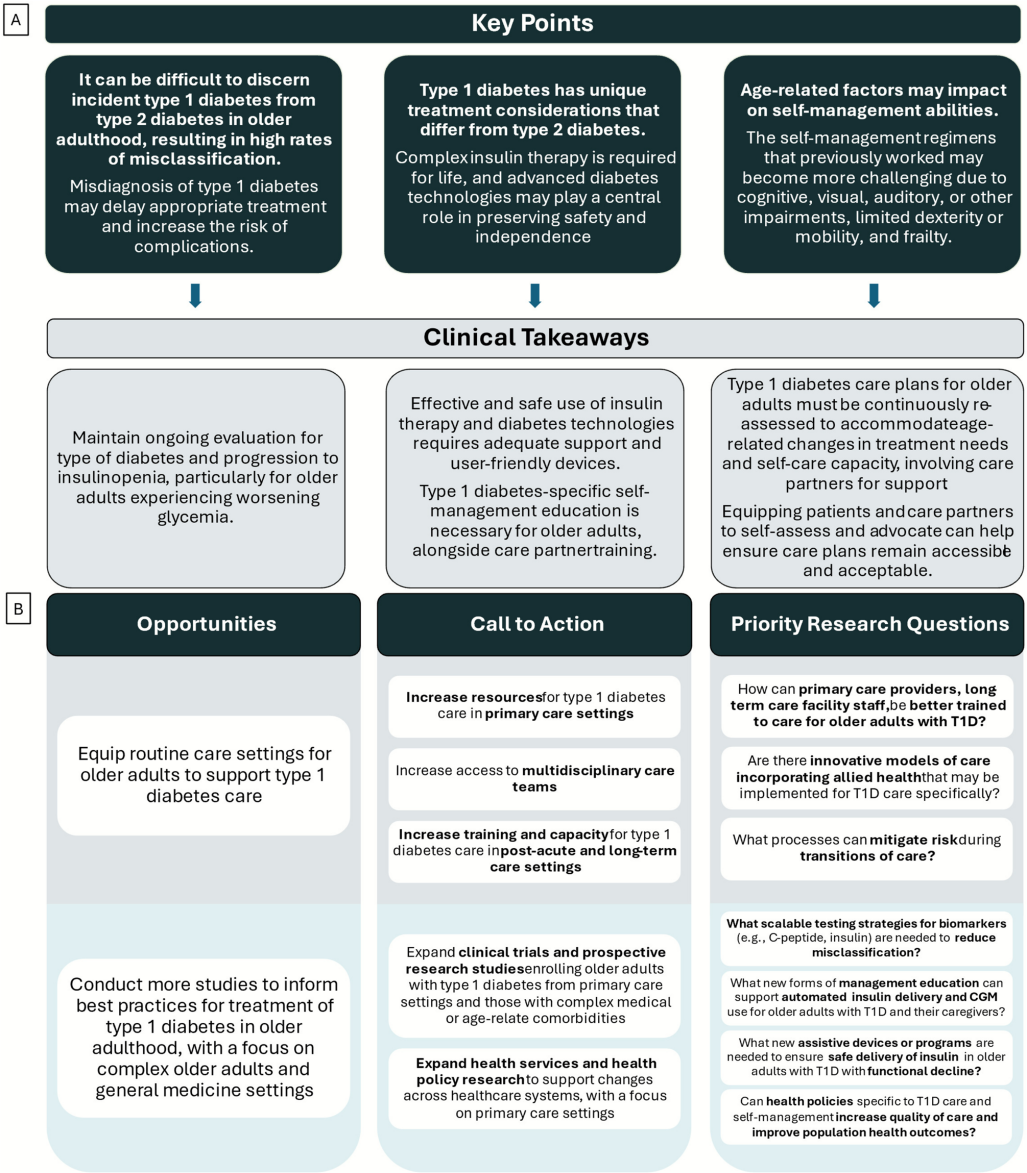
Current knowledge and opportunities to improve care for older adults with type 1 diabetes. (**A**) Summary of key points and clinical takeaways; (**B**) Opportunities, calls to action, and priority research questions.

As previously noted, the current lack of high-quality evidence and guidance specific to the unique needs of older adults with T1D pose challenges to high quality T1D care for older adults across settings. Herein, our multidisciplinary authorship team offers our opinions on current clinical challenges, pertinent unique clinical needs for this populations, and key evidence gaps requiring future study. Figure 2A overviews the clinical challenges and unique needs of this population, and Fig. 2B highlights the timely opportunities to continue to modify care settings and advance evidence for high-quality care and policy.

### Implications for Primary Care Settings

The growing number of older adults living with T1D highlights both the current and future shortage of endocrinologists, geriatricians, and diabetes specialists to provide specialized care for people on complex insulin therapy regimens and using advanced technologies. In the US, many older adults do not have access to specialized diabetes care, with just under one third of Medicare fee-for-service beneficiaries with T1D receiving endocrinology care in recent years, and even lower utilization of diabetes education.^75^

While T2D management is a core component of residency training for family practice and internal medicine, T1D management often receives less attention due to its lower prevalence. Combined with less specialized support for T1D management (e.g., lack of diabetes educators, nutritionists, diabetes technology support teams, etc.), general practitioners may not feel well-equipped or supported to deliver optimal T1D care to older adults in their clinic settings.

Emerging specialized providers, nursing and pharmacist post-graduate training programs, use of telemedicine, and innovative multi-specialty provider networks may provide solutions to the limited availability of specialized care and resources for older adults with T1D. A key challenge for policy reform is the relatively low prevalence of older adults with T1D in most primary care settings, raising the question of whether it is practical to expect all providers to maintain advanced expertise and infrastructure for intensive management. Instead, policy efforts may be more effective if focused on defining core competencies and establishing clear referral pathways to specialist care.

### Priorities for the Future

Healthcare system changes may help to better equip routine care settings for older adults to support T1D care, including resources for increasing access to subspeciality care teams (e.g., endocrinologists, pharmacists, dietitians, and Certified Diabetes Care and Education Specialists). Additionally, with the rapid advancement of diabetes technologies capable of improving care and outcomes in these settings, there is a large gap in evidence and infrastructure to support technology use, including integrating technology use into training and continuing medical education initiatives and processes to improve safety during care transitions.

Providing more support and enhancing staff and practitioner competencies across care settings, such as hospitals, rehabilitation centers, and skilled nursing facilities, will also be necessary to better accommodate older adults with T1D, including focused risk mitigation during transitions of care. Medical alerts and best practice advisories may ensure safer management across care settings, particularly when prescribing insulin. With older adults with T1D represent a rapidly growing population receiving care in acute and extended care facilities, studies informing care and best practices in these settings is very limited.

Because many clinical trials that have advanced the standard of care in T1D have excluded clinically complex older adults with multimorbidity, as well as older adults who receive their T1D care in primary care, more research is needed to characterize empirical differences in care regimens (e.g., pharmacotherapy and diabetes technology approaches) and outcomes across care settings and to stratify older adults, including identifying those for whom specialty care should be prioritized.

Research to support changes across healthcare systems and at the policy level, in combination with education and multi-specialty collaboration, will ensure that healthcare providers and their healthcare systems are equipped and prepared to better meet the needs of the growing number of older adults with T1D.

## Supplementary Information

Below is the link to the electronic supplementary material.

Supplementary file1 (DOCX 17 KB)
