# Supplementary material for: Preparing to Meet the Needs of a Growing Older Adult Population with Type 1 Diabetes: A Narrative Review
Source: J Gen Intern Med. 2025 Dec 8;41(4):1116–28. doi: 10.1007/s11606-025-10053-3 (PMC13004274; doi:10.1007/s11606-025-10053-3)
Supplement: Supplementary file 1 — Supplementary file1 (DOCX 17 KB) [file 11606_2025_10053_MOESM1_ESM.docx]

APPENDIX: Supplemental Material

| Table S1: Search terms for literature review^1^ | |
| --- | --- |
| Focus: Qualitative studies | (("older adults"[Title/Abstract] OR "aged"[MeSH Terms] OR "elderly"[Title/Abstract] OR "seniors"[Title/Abstract] OR "senior citizens"[Title/Abstract]) AND ("type 1 diabetes"[Title/Abstract] OR "diabetes mellitus, type 1"[MeSH Terms] OR "diabetes type 1"[Title/Abstract] OR "diabetes mellitus insulin dependent"[Title/Abstract] OR "type 1 diabetes"[Title/Abstract] OR "diabetes type 1"[Title/Abstract] OR "diabetes mellitus insulin dependent"[Title/Abstract] OR "diabetes mellitus insulin dependent"[Title/Abstract] OR "insulin dependent diabetes mellitus"[Title/Abstract] OR "diabetes mellitus juvenile onset"[Title/Abstract] OR "diabetes mellitus juvenile onset"[Title/Abstract] OR "juvenile onset diabetes mellitus"[Title/Abstract] OR "IDDM"[Title/Abstract] OR "diabetes mellitus type i"[Title/Abstract] OR (("diabetes mellitus"[MeSH Terms] OR ("Diabetes"[All Fields] AND "Mellitus"[All Fields]) OR "diabetes mellitus"[All Fields]) AND "Sudden-Onset"[Title/Abstract]) OR (("diabetes mellitus"[MeSH Terms] OR ("Diabetes"[All Fields] AND "Mellitus"[All Fields]) OR "diabetes mellitus"[All Fields]) AND "Sudden-Onset"[Title/Abstract]) OR "sudden onset diabetes mellitus"[Title/Abstract] OR "type 1 diabetes mellitus"[Title/Abstract] OR (("diabetes mellitus, type 1"[MeSH Terms] OR "type 1 diabetes mellitus"[All Fields] OR ("Diabetes"[All Fields] AND "Mellitus"[All Fields] AND "Insulin"[All Fields] AND "Dependent"[All Fields]) OR "diabetes mellitus insulin dependent"[All Fields]) AND "1"[Title/Abstract]) OR "insulin dependent diabetes mellitus 1"[Title/Abstract] OR "insulin dependent diabetes mellitus 1"[Title/Abstract] OR "juvenile onset diabetes"[Title/Abstract] OR "diabetes juvenile onset"[Title/Abstract] OR "juvenile onset diabetes"[Title/Abstract] OR "diabetes autoimmune"[Title/Abstract] OR "autoimmune diabetes"[Title/Abstract] OR "diabetes mellitus brittle"[Title/Abstract] OR "brittle diabetes mellitus"[Title/Abstract] OR (("diabetes mellitus"[MeSH Terms] OR ("Diabetes"[All Fields] AND "Mellitus"[All Fields]) OR "diabetes mellitus"[All Fields]) AND "Ketosis-Prone"[Title/Abstract]) OR (("diabetes mellitus"[MeSH Terms] OR ("Diabetes"[All Fields] AND "Mellitus"[All Fields]) OR "diabetes mellitus"[All Fields]) AND "Ketosis-Prone"[Title/Abstract]) OR "ketosis prone diabetes mellitus"[Title/Abstract]) AND ("qualitative"[Title/Abstract] OR "themes"[Title/Abstract])) NOT (diabetes mellitus, type 2 [mesh]) |
| Focus: Cognitive impairment | (((Older adults [tiab] OR aged [mesh] OR elderly [tiab] OR seniors [tiab] OR senior citizens [tiab]) AND (Type 1 diabetes [tiab] OR diabetes mellitus, type 1 [mesh] OR diabetes, type 1 [tiab] OR diabetes mellitus, insulin dependent [tiab] OR Type 1 Diabetes [tiab] OR Diabetes, Type 1 [tiab] OR Diabetes Mellitus, Insulin-Dependent [tiab] OR Diabetes Mellitus, Insulin Dependent [tiab] OR Insulin-Dependent Diabetes Mellitus [tiab] OR Diabetes Mellitus, Juvenile-Onset [tiab] OR Diabetes Mellitus, Juvenile Onset [tiab] OR Juvenile-Onset Diabetes Mellitus [tiab] OR IDDM [tiab] OR Diabetes Mellitus, Type I [tiab] OR Diabetes Mellitus, Sudden-Onset [tiab] OR Diabetes Mellitus, Sudden Onset [tiab] OR Sudden-Onset Diabetes Mellitus [tiab] OR Type 1 Diabetes Mellitus [tiab] OR Diabetes Mellitus, Insulin-Dependent, 1 [tiab] OR Insulin-Dependent Diabetes Mellitus 1 [tiab] OR Insulin Dependent Diabetes Mellitus 1 [tiab] OR Juvenile-Onset Diabetes [tiab] OR Diabetes, Juvenile-Onset [tiab] OR Juvenile Onset Diabetes [tiab] OR Diabetes, Autoimmune [tiab] OR Autoimmune Diabetes [tiab] OR Diabetes Mellitus, Brittle [tiab] OR Brittle Diabetes Mellitus [tiab] OR Diabetes Mellitus, Ketosis-Prone [tiab] OR Diabetes Mellitus, Ketosis Prone [tiab] OR Ketosis-Prone Diabetes Mellitus [tiab]))) AND (Cognitive impairment OR Cognitive dysfunction [mesh] OR Cognitive Dysfunctions [tiab] OR Dysfunction, Cognitive [tiab] OR Dysfunctions, Cognitive [tiab] OR Cognitive Disorder [tiab] OR Cognitive Disorders [tiab] OR Disorder, Cognitive [tiab] OR Disorders, Cognitive [tiab] OR Cognitive Impairments [tiab] OR Cognitive Impairment [tiab] OR Impairment, Cognitive [tiab] OR Impairments, Cognitive [tiab] OR Mild Cognitive Impairment [tiab] OR Cognitive Impairment, Mild [tiab] OR Cognitive Impairments, Mild [tiab] OR Impairment, Mild Cognitive [tiab] OR Impairments, Mild Cognitive [tiab] OR Mild Cognitive Impairments [tiab] OR Cognitive Decline [tiab] OR Cognitive Declines [tiab] OR Decline, Cognitive [tiab] OR Declines, Cognitive [tiab] OR Mental Deterioration [tiab] OR Deterioration, Mental [tiab] OR Deteriorations, Mental [tiab] OR Mental Deteriorations [tiab]) |
| Focus: Frailty | (((Older adults [tiab] OR aged [mesh] OR elderly [tiab] OR seniors [tiab] OR senior citizens [tiab]) AND (Type 1 diabetes [tiab] OR diabetes mellitus, type 1 [mesh] OR diabetes, type 1 [tiab] OR diabetes mellitus, insulin dependent [tiab] OR Type 1 Diabetes [tiab] OR Diabetes, Type 1 [tiab] OR Diabetes Mellitus, Insulin-Dependent [tiab] OR Diabetes Mellitus, Insulin Dependent [tiab] OR Insulin-Dependent Diabetes Mellitus [tiab] OR Diabetes Mellitus, Juvenile-Onset [tiab] OR Diabetes Mellitus, Juvenile Onset [tiab] OR Juvenile-Onset Diabetes Mellitus [tiab] OR IDDM [tiab] OR Diabetes Mellitus, Type I [tiab] OR Diabetes Mellitus, Sudden-Onset [tiab] OR Diabetes Mellitus, Sudden Onset [tiab] OR Sudden-Onset Diabetes Mellitus [tiab] OR Type 1 Diabetes Mellitus [tiab] OR Diabetes Mellitus, Insulin-Dependent, 1 [tiab] OR Insulin-Dependent Diabetes Mellitus 1 [tiab] OR Insulin Dependent Diabetes Mellitus 1 [tiab] OR Juvenile-Onset Diabetes [tiab] OR Diabetes, Juvenile-Onset [tiab] OR Juvenile Onset Diabetes [tiab] OR Diabetes, Autoimmune [tiab] OR Autoimmune Diabetes [tiab] OR Diabetes Mellitus, Brittle [tiab] OR Brittle Diabetes Mellitus [tiab] OR Diabetes Mellitus, Ketosis-Prone [tiab] OR Diabetes Mellitus, Ketosis Prone [tiab] OR Ketosis-Prone Diabetes Mellitus [tiab]))) AND (frailty [mesh] OR Frailties [tiab] OR Frailness [tiab] OR Frailty Syndrome [tiab] OR Debility [tiab] OR Debilities [tiab]) |
| 1 Literature review conducted November 2024 to summarize peer-reviewed literature. Abstracts, books, and white papers were excluded. | |

| Table S2: Manuscripts retrieved from each literature review | | | |
| --- | --- | --- | --- |
| **Focus area** | **Full-text articles returned** | **Excluded for lack of relevance** | **Reviewed and included in the narrative review** |
| Qualitative studies | 97 | 91 | 6 |
| Cognitive impairment | 76 | 56 | 20 |
| Frailty | 9 | 7 | 2 |
| **Total** | **182** | **154** | **28** |
